# Synchrotron‐Based Nano‐X‐Ray Absorption Near‐Edge Structure Revealing Intracellular Heterogeneity of Iron Species in Magnetotactic Bacteria

**DOI:** 10.1002/smsc.202100089

**Published:** 2021-12-23

**Authors:** Daniel M. Chevrier, Elisa Cerdá-Doñate, Yeseul Park, Fernando Cacho-Nerin, Miguel Gomez‐Gonzalez, René Uebe, Damien Faivre

**Affiliations:** ^1^ CNRS CEA BIAM Aix-Marseille Université 13108 Saint-Paul-lez-Durance France; ^2^ Department of Biomaterials Max Planck Institute of Colloids and Interfaces 14476 Potsdam Germany; ^3^ Diamond Light Source Diamond House Harwell Campus, Chilton Didcot OX11 0DE UK; ^4^ Department of Microbiology University of Bayreuth 95440 Bayreuth Germany

**Keywords:** biomineralization, magnetite, magnetosomes, magnetotactic bacteria, nano-X-ray absorption near-edge structure, single cells, X-ray microscopy

## Abstract

Magnetotactic bacteria (MTB) sequester iron from the environment to biomineralize magnetite or greigite nanoparticles in magnetosome organelles, though the necessity of intracellular iron storage for the formation process is still in question. Understanding the role of iron storage would make clear the contribution of MTB in geochemical iron cycling and its potential importance during the biosynthesis of application‐relevant magnetic nanoparticles. Herein, how scanning X‐ray fluorescence microscopy (SXFM) and nanoscale X‐ray absorption near‐edge structure (nano‐XANES) mapping can spatially and chemically identify intracellular iron species is reported, creating an opportunity to examine the role of iron storage in magnetite biomineralization at the single‐cell level. Fe K‐edge nano‐XANES measurements of *Magnetospirillum gryphiswaldense* in varied iron media conditions and iron storage capacity reveal a significant quantity of intracellular iron heterogeneities through a distinction between formed magnetosomes and intracellular iron material. This intracellular iron component is found in both early and late stages of biomineralization. The capabilities of nano‐XANES in providing an experimental advantage in the multidisciplinary field of biomineralization are highlighted.

## Graphical abstract

Scanning X‐ray fluorescence microscopy (SXFM) and nanoscale X‐ray absorption near‐edge structure (XANES) mapping distinguish fully formed magnetite nanoparticles from intracellular iron storage. The differentiation of iron species accomplished by hard X‐ray microscopy offers a new approach to understand magnetite biomineralization in magnetotactic bacteria at the single‐cell level.

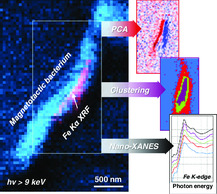

## 1 Introduction

Recent breakthroughs in X‐ray microscopy have enabled new means to investigate the structure and composition of materials ranging from energy‐storage materials to individual cells to nanomaterials.^[^ ^1^, ^2^, ^3^, ^4^, ^5^ ^]^ In particular, X‐ray nanoprobe beamlines at synchrotron facilities provide sub‐100 nm beam sizes in the hard X‐ray energy regime (i.e., 5−30 keV) that enable investigations of the nanoscopic composition, structure, and organization of inorganic constituents in heterogeneous environments through an element‐specific perspective.^[^ ^6^, ^7^, ^8^, ^9^, ^10^ ^]^ The advantage of hard X‐ray nanoprobes is the combination of imaging and spectroscopy, where each pixel or position on the sample contains spectral information at the nanoscale (e.g., X‐ray fluorescence [XRF], X‐ray absorption spectroscopy [XAS], X‐ray diffraction, or scattering). Scanning X‐ray fluorescence microscopy (SXFM) combined with such spectroscopy techniques has been highly advantageous for studying biological samples, revealing novel information on the intracellular biochemistry and role of metals in cells.^[^ ^11^ ^]^ Such X‐ray nanoprobe advancements have investigated intracellular elemental composition and structure of algae,^[^ ^8^, ^12^, ^13^ ^]^ bacteria,^[^ ^4^ ^]^ plants,^[^ ^14^ ^]^ and even small animals.^[^ ^15^ ^]^ Considering these milestones in nanoscale X‐ray imaging of organisms, few studies have employed these approaches to address biological questions directly.

Microorganism‐controlled biomineralization represents one of the most sophisticated means to produce inorganic nanomaterials. Magnetotactic bacteria (MTB) exemplarily synthesize single‐domain magnetic nanoparticles composed of magnetite (Fe_3_O_4_) or greigite (Fe_3_S_4_) within organelles known as magnetosomes. Magnetosomes are formed by a highly controlled biological mechanism, where the number, morphology, and arrangement of magnetic nanoparticles are species specific.^[^ ^16^, ^17^, ^18^, ^19^ ^]^ The mechanism behind magnetite biomineralization in MTB is still unclear, with much left to understand in terms of iron uptake, iron storage, nanocrystal nucleation, and growth.^[^ ^20^ ^]^ X‐ray absorption fine structure (XAFS), Mössbauer spectroscopy, and electron microscopy have been invaluable techniques to reveal initial insights into the biosynthesis of magnetite. Together, they have identified several iron oxide phases and iron precursors potentially involved in the formation mechanism (e.g., ferrihydrite, high‐spin Fe^2+^ complexes, ferritin proteins, hematite, phosphate‐rich ferric hydroxide).^[^ ^21^, ^22^, ^23^, ^24^ ^]^ More recently and relevant to the geochemical influence MTB have on iron cycling, studies have identified a large fraction of intracellular iron, at least partly composed of ferrous species, that is not incorporated into magnetosomes.^[^ ^25^, ^26^, ^27^ ^]^ The implications and localization of this stored iron are under question: whether it is pertinent to magnetite formation and if it is mainly found within magnetosome membranes,^[^ ^26^, ^28^ ^]^ contained by ferritin proteins,^[^ ^29^ ^]^ or a more labile source in the cytoplasm.^[^ ^26^, ^28^ ^]^

With these biological questions in the forefront, X‐ray spectromicroscopy techniques could lead to a clearer understanding of magnetosome formation by discerning iron species in distinct intracellular regions (e.g., membrane, cytoplasm, magnetosome chain), thereby avoiding convoluted chemical structural information from bulk measurements. Scanning transmission X‐ray microscopy (STXM), which operates in the soft X‐ray regime, has been employed recently to follow magnetosome formation and organization on the single‐cell level, with additional information on the changing valence of iron and the evolution of magnetic properties using X‐ray magnetic circular dichroism.^[^ ^30^, ^31^ ^]^ However, when probing the Fe L_2,3_‐edge (700−730 eV) with STXM, the short core‐hole lifetime limits the collection of local structural information via X‐ray backscattering processes. Higher‐energy Fe K‐edge XAFS (7100−7700 eV) would be advantageous in this regard by affording longer core‐hole lifetimes, which create more distinctive XAFS features that could help define unknown iron species (e.g., amorphous precursors, phosphate‐rich ferric hydroxides). In addition, SXFM is highly suitable for detecting trace‐level metal species as XRF events are directly measured.

Herein, we demonstrate how hard X‐ray microscopy can be used as a new approach to probe magnetosome formation at the single‐cell level via nano‐XRF mapping and nano‐X‐ray absorption near‐edge structure (XANES) analysis. We measured wild‐type (WT) *M. gryphiswaldense* MSR‐1 bacteria and a genetic variant lacking ferritin proteins under varied iron concentrations and at different stages of magnetosome formation. Dark‐field differential phase contrast (DPC) X‐ray imaging was first coupled with nano‐XRF mapping to reconstruct both organic and inorganic components of bacterial cells to discern intracellular regions. Through principal components, linear combination (LC) fitting, and cluster analyses of Fe K‐edge nano‐XANES data, we then differentiated magnetosome particles from other intracellular iron species and determined their relative amounts. This study confirms a significant presence of intracellular iron species distinct from magnetite during biomineralization by examining varied iron media concentrations and early/late stages of formation.

## 2 Results

### 2.1 Scanning X‐ray Fluorescence Imaging of MTB

**Figure 1:**
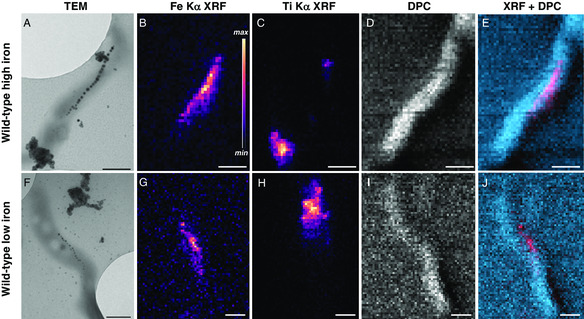
Electron and XRF microscopy of MTB. Individual MTB (WT *M. gryphiswaldense*) grown under standard conditions (first row, “WT high iron”) and under iron‐limited conditions (second row, “WT low iron”) measured with A,F) TEM and B−E) and G−J) SXFM. D,I) X‐ray scattering was collected in transmission to produce DPC maps to image cellular membrane material. E,J) Composite maps of Fe Kα (red) and DPC (blue). Incident X‐ray energy was 9 keV with −50 to 60 nm X‐ray beam diameter step size of 50 nm. Scale bars: 500 nm.

Individual *M. gryphiswaldense* cells were measured with SXFM using the X‐ray nanoprobe beamline (I‐14) at Diamond Light Source ^[^ ^10^ ^]^ and then with transmission electron microscopy (TEM) to confirm the relative location of magnetosomes and cell membrane. **Figure** **1** displays bacteria characterized with both microscopy techniques (TEM measurements followed SXFM), presented in the same orientation. With an X‐ray beam size of ≈50−60 nm, approaching that of the fully formed magnetosomes in Figure 1A (magnetosome diameter = 42 ± 7 nm [*n* = 24] and diameter range = 25−56 nm), the Fe Kα XRF signal (Figure 1B) displays the magnetosome chain configuration, as seen from the TEM image (see Figure S1, Supporting Information, for XRF spectrum example). Even for smaller magnetosome particles, such as those for *M. gryphiswaldense* grown under iron‐limited conditions in Figure 1F (magnetosome diameter = 24 ± 8 nm [*n* = 27], diameter range = 10–37 nm), the Fe Kα XRF signal (Figure 1G) resembles the distribution of magnetosomes and orientation of the magnetosome chain (Table S1, Supporting Information, lists the samples studied herein and their respective conditions). We also used X‐rays scattered by the bacterium to produce DPC images. Combining XRF and DPC signals produces a composite map that replicates the image seen from TEM (see Figure 1A/E,1F/J), enabling assessment of iron species and their intracellular location from SXFM data alone.

Though the spatial resolution of SXFM is unable to match that of TEM, this hard X‐ray approach enables collection of Fe K‐edge XAFS over the entire cell region, offering a new spectral vantage of chemical speciation at the single‐cell level for MTB. A stack of XRF maps collected at varied energies across the Fe K‐edge (i.e., 151 maps for 151 energy points) were combined and aligned to produce a stack or 2D XAFS data, where each pixel translates to an XAFS spectrum. XAFS data was truncated to show only the near‐edge region due to weak postedge oscillations; therefore, datasets are further referred to as nano‐XANES.^[^ ^15^ ^]^ To improve the tracking and alignment between each map during measurement and postprocessing, we deposited TiO_2_ nanoparticles onto the substrate to provide a reference point for the stack alignment procedure (see Figure 1A/C,F/H). The analysis of nano‐XANES from four different samples in various states of biomineralization is presented below. One limitation of the nano‐XANES approach is the time required to complete a stack of maps. Even though the Ti Kα fluorescence aided in realignment of the scanning region between maps to counter drifting issues, several hours are still required to acquire a complete nano‐XANES dataset. As a result, nano‐XANES of only one cell was collected for each condition.

### 2.2 Nano‐XANES Analysis of Magnetosome Formation

**Figure 2:**
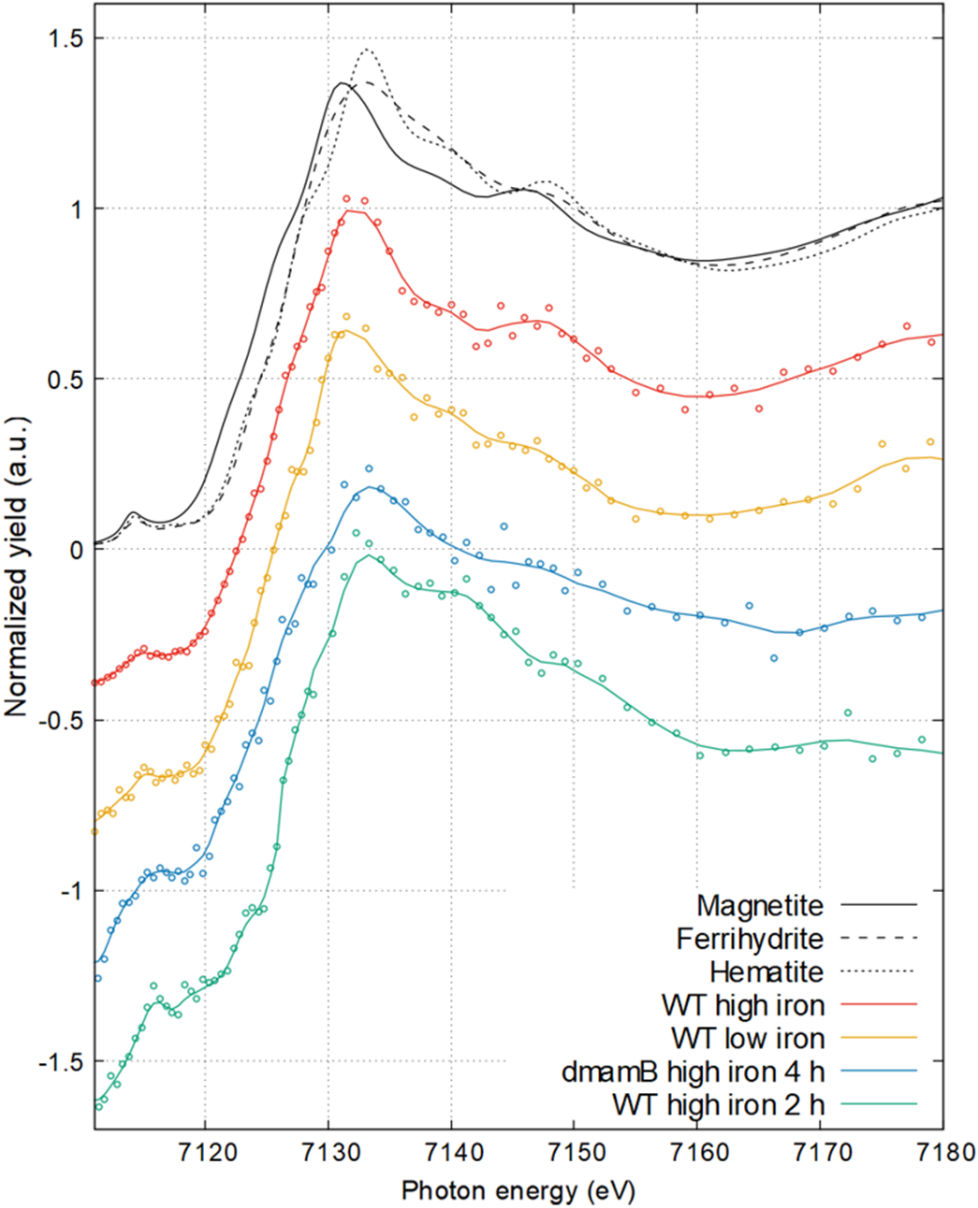
XANES of individual MTB. Fe K‐edge XANES of reference materials and of the full‐cell region for samples of *M. gryphiswaldense*: WT high iron, WT low iron, Δ*mamB* high iron 4 h, and WT high iron 2 h.

We first consider the nano‐XANES dataset for WT *M. gryphiswaldense* grown under standard conditions, where 50 μM Fe(III)−citrate is added to ensure complete magnetosome formation (sample referred to as “WT high iron”). The Fe K‐edge XANES signal from the entire cell region is presented in **Figure** **2** with some iron oxide references for comparison and the other samples to be investigated herein (see Section 2.3 on early‐stage biomineralization). A positive shift in absorption edge energy is observed for WT high iron compared with magnetite (Δ*E*_0−0mag_ = *E*_0_ − *E*_0magnetite_) of +1.0 eV. Also found is the broadening of the white line (most intense absorption feature between 7130 and 7135 eV) and the near‐edge feature around 7145 eV. The LC fitting results yielded the following contributions: 60% magnetite (Fe_3_O_4_), 33% hematite (Fe_2_O_3_), and 7% goethite (FeO(OH)) (*R*‐factor = 0.0011); ferrihydrite (Fe_2_O_3_ • 0.5H_2_O), phosphate‐rich ferric hydroxides, ferric chloride (FeCl_3_), and ferrous chloride (FeCl_2_) were also included as references in combinatorial fitting (see Figure S2, Supporting Information). LC fitting results are summarized in Table S2, Supporting Information, with XANES fits in Figure S3–S6, Supporting Information. The high amount of the ferric material could originate from a combination of magnetite oxidation, ferric iron storage, ferritin proteins, and ferrous iron originally stored in the cell oxidized from sample preparation and ambient measurement conditions. We caution that herein LC fitting results help interpret the relative composition of intracellular iron species and discern these from magnetosome particles but should not be taken directly for the actual composition. This is due to the XAFS measurement of nanostructured and amorphous materials that yield XANES spectra which do not resemble bulk‐like materials and thus may vary in near‐edge features (e.g., spectral broadening, energy shift).^[^ ^32^, ^33^ ^]^ In addition, drying effects from sample preparation may blur the results.

We utilized nano‐XANES data to elucidate the location of iron species in the cell distinct from magnetite nanoparticles, where each pixel of the XRF map stack contains a XANES spectrum. To expedite the analysis of >1000 XANES spectra per mapped region, we used principal components and cluster analyses to objectively describe the variance in nano‐XANES data, therefore aiding in the differentiation of distinct iron species. Principal components analysis (PCA) of nano‐XANES data calculates eigenspectra and eigenimages from the map stack. Eigenspectra are an orthogonal set of spectra that, in LC, can describe any observed spectrum in the dataset. The eigenvalue associated with each eigenspectrum gives a typical weighting of that eigenspectrum in the entire dataset, where eigenspectra with smaller eigenvalues represent less variations in the data.

**Figure 3:**
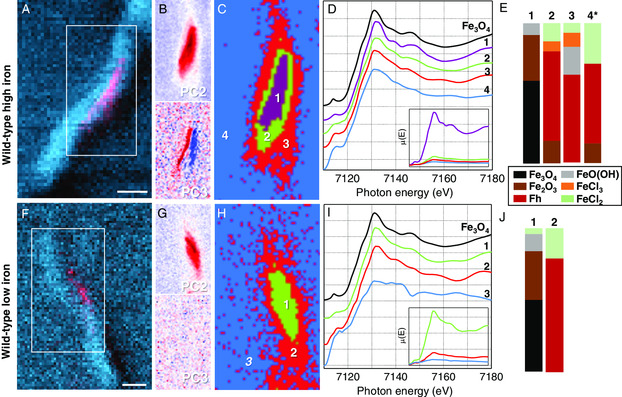
Nano‐XANES data for MTB with complete magnetosome chains. A,F) WT‐high‐iron and WT‐low‐iron nano‐XANES regions (white box), B,G) PCA maps (red signal), C,H) maps of cluster centers, D,I) normalized offset Fe K‐edge XANES spectra (inset with normalized spectra retaining edge jump values), and E,J) a summarized composite of LC fitting results (* indicates *R*‐factor ≥ 0.02 from LC fitting result).

Examining the region indicated in **Figure** **3A** of WT high iron, PCA identified three components or three eigenimages (Figure S7, Supporting Information) that describe the variance in nano‐XANES data. The second component shown in Figure 3B (PC2, red region) accounts for most of the bacterium against the substrate background (where PC1 accounts for the bacterium and the substrate, Figure S7, Supporting Information). The third component (PC3) further distinguishes the magnetosome chain from intracellular iron species, confirmed by the agreement of the magnetosome chain length and orientation in Figure 1A. Cluster analysis was then performed to group nano‐XANES data according to spectral similarities into distinct intracellular regions, where the relative amount and location of distinct Fe K‐edge XANES signals were inspected.

Four cluster centers were sufficient to represent WT‐high‐iron nano‐XANES (i.e., a fifth cluster did not further differentiate the XANES signal). The cluster centers map and corresponding XANES spectra for each cluster are presented in Figure 3, respectively. Cluster 1 encompasses the magnetosome chain, as evidenced by accounting for ≈73% of the total XANES signal intensity, shown by *μ*(*E*) spectra retaining respective signal intensities for each cluster (Figure 3D, inset). The near‐edge structure of cluster 1 resembles the magnetite reference similar to the full‐cell XANES, but again with a positive shift in absorption edge (Δ*E*_0‐0mag_ = +1.1 eV) and broader near‐edge features, suggesting a mixture of components or partial oxidation to a ferric oxide.^[^ ^32^ ^]^ LC fitting results are summarized in Figure 3E, indicating a composition of 59% magnetite, 33% hematite, and 8% goethite (*R*‐factor = 0.0011). Besides partial oxidation of magnetite, we anticipated that iron‐storage proteins such as ferritin and labile iron ions could contribute to intracellular ferric oxide‐fitted components.

LC fitting for cluster 2, which encircles cluster 1 and still within the intracellular region, yielded 16% hematite, 64% ferrihydrite, 7% ferric chloride, and 13% ferrous chloride (*R*‐factor = 0.0042) and accounted for ≈13% of the total XANES signal. We note that although ferric and ferrous chloride were fit to the XANES spectrum of cluster 2, it does not directly indicate that this particular species is present; rather, it likely accounts for more ionic Fe(III) and Fe(II) species. It could also originate from residual growth media that dried from sample preparation leaving salt‐like iron material around the cell. Moreover, the components fitted reflect the iron material in the measured state, not the native state of the cell. Clusters 3 and 4, which represent the perimeter of the intracellular region and the substrate, respectively, have fewer distinguishable near‐edge features than cluster 2 and a combined account for less than 10% of the total XANES signal intensity. A larger portion of ferrihydrite is fitted due to broader XANES features. Cluster 4 is expectantly the weakest signal and gave a higher composition of iron chlorides though with poor goodness of fit, which reflects the dilute substrate background.

The ferric oxide material associated with the magnetosome chain in cluster 1 indicated from LC fitting and measured absorption edge shift suggests that magnetite nanoparticles could be partially oxidized from X‐ray exposure and/or from intracellular iron species that have condensed around the magnetosome chain. The latter is suspected as PC2 (Figure 3B) shows the total intracellular iron XANES signal to be concentrated around the magnetosome chain region and not be dispersed throughout the entire cell.

To address the intracellular iron content and potential magnetite oxidation from hard X‐ray measurement, *M. gryphiswaldense* grown under low‐iron conditions to limit excess iron storage (“WT low iron”, Figure 1F and Table S1, Supporting Information) was inspected with nano‐XANES. Examining the XANES spectrum for the entire cell in Figure 2, again, there are spectral similarities with magnetite and here with a more similar absorption edge position (Δ*E*_0‐0mag_ = +0.4 eV). LC fitting results for WT low iron gave 45% magnetite, 52% ferrihydrite, and 3% ferrous chloride (*R*‐factor = 0.0017). In this case, the higher percentage of ferric material fitted may be caused by spectral broadening from the smaller magnetite nanoparticles (see Figure 1F), which is also evidenced by the weaker near‐edge feature at 7145 eV.

Different from WT high iron, a third PCA component did not account for the magnetosome chain in WT low iron (Figure 3G), where this eigenimage shows random variations of the signal from pixel to pixel. Cluster analysis with three cluster centers represented the concentric diversity of nano‐XANES data outward from the magnetosome chain, shown in Figure 3H,I. Cluster 1 accounts for most of the region depicted in PC2 with ≈75% of the total signal intensity (inset in Figure 3I). Although the absorption edge energy shift is closer to magnetite (+0.2 eV) than cluster 1 for WT high iron, LC fitting results show 50% magnetite, 34% hematite, 12% goethite, and 4% ferrous chloride (*R*‐factor = 0.0016), indicating the presence of ferric oxide material in the magnetosome chain region. Cluster 2 shows a similar absorption edge position to cluster 1 and near‐edge features that are less defined due to the lower signal intensity (≈17% of total signal). This region is comparable with cluster 2 for WT high iron in terms of LC fitting results and absorption edge shift (see Table S2, Supporting Information). The last cluster again represents the substrate region, but the XANES signal is too weak to fit.

Similar compositions of magnetite and ferric oxides were found from cluster analysis and LC fitting results of the magnetosome region for WT high iron and WT low iron (i.e., clusters 1 and 2). We also note that ferrihydrite accompanies magnetite in clusters 2 and 3 similar to what Fdez‐Gubieda et al. found where MSR‐1 samples were measured in a dried state at varied time points in the biomineralization process.^[^ ^23^ ^]^ However, it is still not clear from WT low iron if there remains a significant amount of stored iron in the cell or if the fitted ferric oxide contribution is due to oxidation of magnetite. To help answer this, WT *M. gryphiswaldense* was extracted at early‐stage biomineralization, where higher amounts of intracellular iron storage are expected, to determine if this contribution could be better separated from developing magnetosomes rather than fully formed magnetosome chains. The signal from this intracellular iron was further confirmed by measuring a ferritin‐deficient genetic variant of *M. gryphiswaldense* also at early‐stage biomineralization.

### 2.3 Early‐Stage Magnetosome Formation

**Figure 4:**
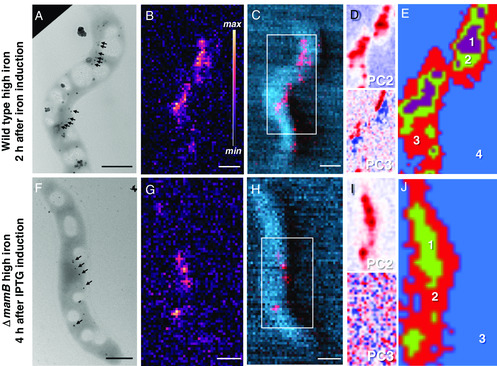
TEM, SXRF, and nano‐XANES of MTB in early‐stage magnetosome formation. *M. gryphiswaldense* WT high iron 2 h (first row) and *ΔmamB* high iron 4 h (second row). A,F) TEM, B,G) Fe Kα XRF maps, and C,H) composite maps of Fe Kα XRF (red) and DPC (blue). Principal component maps for D,I) Fe K‐edge nano‐XANES and E,J) cluster maps of indicated regions in composite maps. Incident X‐ray energy 9 keV for XRF and DPC maps. X‐ray map pixel size 50 nm. Scale bar: 500 nm.

Two *M. gryphiswaldense* samples, WT and a genetic variant (Δ*mamB*Δ*dps/bfr12*::P_lac_ *‐mamB,* see Experimental Section), were extracted from culture media a few hours after initiating magnetosome formation via different mechanisms. The early‐stage biomineralization WT sample was initially cultured under very low levels of iron to suppress magnetosome formation, introduced into iron‐rich media to initiate biomineralization and then extracted after 2 h (sample referred to as “WT high iron 2 h”). **Figure** **4** displays TEM, Fe Kα XRF, and the DPC−XRF composite map for WT high iron 2 h, respectively. Compared with WT high iron and WT low iron, the Fe Kα XRF signal is more dispersed throughout the cell with weaker total signal intensity due to nascent magnetosomes (magnetosome diameter = 21 ± 7 nm [*n* = 9] and diameter range = 11−33 nm). Despite this, two segments of the forming magnetosome chain are still apparent in the XRF map (Figure 4H).

The full‐cell Fe K‐edge XANES for WT high iron 2 h (Figure 2) has an energy shift of +1.6 eV (Δ*E*_0‐0mag_) and a more pronounced near‐edge feature following the white line around 7140 eV. Both indicate a significant amount of disordered ferric species, which has been similarly observed for amorphous ferric iron from dps‐like proteins or phosphate‐rich ferric hydroxides.^[^ ^22^, ^23^ ^]^ Regarding the latter species, Figure S2, Supporting Information, shows this characteristic near‐edge feature. LC fitting was attempted for the full‐cell XANES spectrum but a low goodness of fit (*R*‐factor = 0.032) prevents interpretation of the results (see Table S2, Supporting Information). Nevertheless, as shown in Figure 4D, the PC2 eigenimage presents most of the intracellular iron and PC3 accounts for the two developing magnetosome chain segments (see indicated particles in Figure 4A), similar to PC2 and PC3 for WT high iron (Figure 3B). Four cluster centers were used to separate intracellular XANES signals, as shown in Figure 4E (cluster spectra shown in Figure S8, Supporting Information). Like WT high iron, cluster 1 accounts for the magnetosome chain regions and clusters 2 and 3 for the intracellular region proximal to magnetosomes.

The genetic variant (referred to as Δ*mamB* for short), the second early‐stage biomineralization sample, is unable to form magnetosome membranes due to lack of the gene encoding for the *MamB* protein, which contributes to the structure of encapsulating membranes.^[^ ^34^ ^]^ Δ*mamB* also lacks iron‐storage proteins (e.g., ferritin family proteins) that act as iron reservoirs. This variant was designed so that magnetosome formation can be triggered via IPTG, a gene expression inducer (see Experimental Section), to activate the *mamB* gene and resume magnetosome formation. TEM image, Fe Kα XRF map, and DPC−XRF composite map are shown in Figure 4F−H for a cell that was extracted from culture after 4 h of post‐IPTG induction (sample referred to as “Δ*mamB* high iron 4 h.” See Experimental Section for explanation of 4 h time point). More discrete Fe Kα XRF signals are observed compared with WT samples (see Figure 4G) from the few magnetosomes (magnetosome diameter = 21 ± 8 nm [*n* = 5] and diameter range = 11−30 nm) and lack of ferritin material, which greatly reduces intracellular iron during early biomineralization.

Due to the weaker Fe Kα XRF signal of few nascent magnetosomes, nano‐XANES cluster spectra are of lower quality for early‐stage biomineralization (Figure S8, Supporting Information) and hence LC fitting results are again less reliable than for WT samples with complete magnetosome chains. The main‐edge shoulder and white‐line position are comparable with WT high iron, though the postedge feature around 7145 eV is absent likely from the weaker signal and smaller magnetosomes. Comparing the full‐cell XANES for both early‐stage formation samples (Figure S9, Supporting Information), a positive shift of the main absorption edge and increased intensity of the near‐edge feature around 7140 eV (see phosphate‐rich ferric hydroxide, Figure S2, Supporting Information) for WT high iron 2 h further support the higher amount of intracellular iron species that is likely in reserve from the iron‐induction experiment and absent for Δ*mamB* high iron 4 h. PCA shows two components that describe the variance in the data (Figure 4I and S7, Supporting Information) with the second eigenimage being discrete magnetosome particles, indicating low amounts of intracellular iron aside from magnetosomes. Three cluster centers separate intracellular regions (Figure 4J and S8, Supporting Information), though their LC fitting results show poor goodness of fit. Considering both early‐stage biomineralization samples, PCA and cluster analysis still identify irreducible components that distinguish developing magnetosomes from intracellular iron species likely from ferritin‐type iron storage.

## 3 Discussion

The contribution of intracellular iron storage to the XANES signal at early‐stage formation could be assessed from a comparison of WT low iron 2 h and Δ*mamB* high iron 4 h, where the full‐cell XANES of the latter (Figure S9, Supporting Information) indicate less ferric oxide material (i.e., lower Δ*E*_0‐0mag_) and with more features similar to magnetite. This further suggests that a significant amount of ferric species detected as ferric oxides in WT samples is likely from iron storage or oxidation of intracellular ferrous species (e.g., Fe(II) in cytoplasm or membrane structures that was oxidized during sample preparation/measurement) and not only from oxidation of magnetite. Thus, it is anticipated that this intracellular iron pool is not completely depleted with a detectable amount left after full magnetosome chain formation.

The presence of stored iron (i.e., labile and protein bound) is not a surprise but its persistence after biomineralization is, as it may have complicated or eluded previous interpretations of time‐dependent magnetosome formation studies. From time‐course XAS studies with bulk samples, the appearance of magnetite nanoparticles in MTB appears to quickly outweigh the contribution of intracellular ferric and ferrous species in the X‐ray absorption signal collected.^[^ ^22^, ^23^, ^24^ ^]^ Therefore, amorphous or precursor iron species that were detected at early time points were thought to feed or precede magnetite formation, though it may persist throughout the entire biomineralization process, as shown in this work. Recent studies with ferritin‐suppressed mutants suggest that another mechanism may be at work when these proteins are absent and that, accordingly, they may not be essential for biomineralization.^[^ ^29^, ^34^ ^]^ This possibility highlights the plasticity of biological processes with the bacteria making use of intracellular material (i.e., ferritin) when it is present and utilizing an alternative predeveloped pathway when it is absent.

For WT samples, iron storage species appear to agglomerate around the magnetosome chain, potentially from ferritin material that condenses onto magnetosome particles during the sample drying process. This is particularly evident for WT high iron 2 h, where intracellular iron is found segmented according to the two portions of the developing magnetosome chain (Figure 4). Although less ideal for capturing native‐state information of the cell, drying effects from sample preparation may concentrate intracellular iron into certain regions (e.g., magnetosome chain), leading to a higher intensity XANES signal. Considering XANES signal intensity (XANES edge jump from XRF signal) from intracellular cluster regions around the magnetosome chain (e.g., cluster 2 or clusters 2 and 3, regions outside the magnetosome chain but within the cell membrane), around 22%, 16%, 48%, and 28% is intracellular iron besides magnetite for WT high iron, WT low iron, WT high iron 2 h, and Δ*mamB* high iron 4 h, respectively. This still excludes additional iron species that are likely covering magnetosomes in cluster 1 and indistinguishable by the measurement. In fact, recent studies have reported similar substantial intracellular iron content separate from magnetosomes.^[^ ^25^, ^26^, ^27^ ^]^ In separate studies, Berny et al. and Amor et al. found that only ≈30–50% of total intracellular iron content from both fully grown MSR‐1 and *Magnetospirillum magneticum* (AMB‐1) cultures was used to produce magnetite. Amor et al. went on to characterize intracellular iron speciation and subcellular localization using fluorescence assays with AMB‐1. Furthermore, they determined that magnetite had only accounted for ≈30% of intracellular iron. This was further confirmed by a delay in magnetite precipitation as iron internalization increases.

Together, SXFM and nano‐XANES bridge the gap between high‐spatial‐resolution single‐cell imaging (e.g., HRTEM) and high‐spectral‐resolution bulk characterization (e.g., scanning XAFS). This combined approach has the potential to recover more information on a cell‐to‐cell basis from a single experiment by combining adequate imaging and spectral resolution, enabling opportunities to make new progress in understanding biomineralization processes. Further improvements in X‐ray nanoprobe instrumentation and measurements are expected with the availability of more fourth‐generation synchrotron radiation facilities, offering brighter X‐ray beams and higher coherence. For future work on magnetite biomineralization in MTB, conducting SXFM and nano‐XANES under cryogenic conditions will enable vitrified samples to be measured to preserve iron homeostasis and reduce beam‐induced changes to the sample. Another advantage of using hard X‐ray spectromicroscopy to study biomineralization processes is the emerging capabilities of integrated microfluidic cell sample environments to examine dynamic processes such as iron uptake and sequestration in situ.

## 4 Conclusion

Shown in this work, the intracellular iron heterogeneity of individual MTB can be inspected with a semiautomated treatment of Fe K‐edge nano‐XANES data to reveal single‐cell‐level information on iron homeostasis related to the biosynthesis of magnetite nanoparticles. Early‐stage biomineralization samples illustrate how nascent magnetosomes can be distinguished from iron storage species by PCA and cluster analysis. Although it is difficult to elucidate the exact chemical nature of iron species due to their amorphous and nanostructured nature, plus mixing and drying effects, our series of samples show how ferritin, a main source of iron‐protein storage in *M. gryphiswaldense,* persists from early biomineralization to complete magnetosome chain formation. Investigating intracellular biomineralization with Fe K‐edge nano‐XANES sets a precedent for the level of combined chemical imaging information that is now achievable on the single‐cell level with hard X‐ray nanoprobe measurements.

## 5 Experimental Section

5.1

5.1.1

##### Culture Media Conditions, Genetic Variants, and Sample Preparation

*M. gryphiswaldense* (MSR‐1) was grown under microaerobic conditions in septum‐stoppered glass tubes (1% oxygen) in a modified flask standard medium (FSM) for cultured MTB.^[^ ^35^ ^]^ *M. gryphiswaldense* collected after at least 72 h of growth was referred to as “WT high iron.” *M. gryphiswaldense* was also grown in modified low‐iron‐media conditions (no Fe(III)−citrate added to FSM), where samples collected after 72 h of growth were referred to as “WT low iron.” For magnetosome induction experiments, *M. gryphiswaldense* was first grown in modified FSM medium with very low iron (no Fe(III)−citrate and no peptone) and under aerobic conditions for at least two passages. In the third passage, cultures were transferred at the early logarithmic phase to FSM (high‐iron conditions) and under microaerobic conditions to induce magnetosome formation. An aliquot was removed after 2 h of exposure to iron‐rich media and was referred to “WT high iron 2 h” sample. Alternative to this magnetosome induction method, a genetic variant was used to trigger magnetosome formation via genetic induction. The essential *mamB* gene was deleted within the *M. gryphiswaldense* ferritin genetic variant Δ*dps/bfr12* ^[^ ^29^ ^]^ using the plasmid pORΔ*mamB*.^[^ ^36^ ^]^ This genetic variant was then complemented by site‐specific Tn7 transposon‐mediated chromosomal integration of an isopropyl β‐D‐1‐thiogalacto‐pyranoside (IPTG)‐inducible P_lac_‐*mamB* construct.^[^ ^34^ ^]^ The resulting strain Δ*mamB*Δ*dps/bfr12::*P_*lac*_ *‐mamB* was grown under conditions similar to WT high iron with the exception that 2 mM IPTG was added. A sample was removed 4 h after IPTG induction and not 2 h because of a 1−2 h delay in gene activation. This sample was referred to as “Δ*mamB* high iron 4 h.”

Samples of *M. gryphiswaldense* were taken as small aliquots from culture media, centrifuged to remove culture media, and then washed with 0.01 m HEPES and 0.02 m EDTA buffer (pH 7) three times to remove excess metal ions. Finally, cells were washed once with Milli‐Q water (18.2 Ω m^−1^). 5 μL of washed cells were deposited onto a parafilm with a TEM grid (gold or copper metal with holey carbon film) placed on top of each drop (carbon side down) for at least 30 min. Afterward, the grids were submerged in Milli‐Q water for 10 s three times and then dried on filter paper. 5 μL of a 1/50 000‐diluted TiO_2_ nanoparticle solution (≈150 nm in diameter, 33 wt% TiO_2_ in water, Sigma Aldrich) was added to each TEM grid to provide a measurable secondary XRF signal for tracking and alignment purposes for XANES mapping acquisition of single cells.

##### Hard X‐Ray Nanoprobe Measurements

TEM grids were mounted for measurement at the I‐14 hard X‐ray nanoprobe beamline (Diamond Light Source Ltd., Didcot, UK) using custom holders designed and supplied by the beamline.^[^ ^10^ ^]^ SXFM measurements were conducted under ambient conditions using an incident photon energy of 9 keV for XRF mapping and a range of 7.0−7.3 keV for collection of Fe K‐edge XANES maps. The focused X‐ray beam was 50−60 nm (FWHM) in diameter. XRF from the sample was collected in front of the sample using a four‐element silicon drift detector (RaySpec, UK). A raster scanning step size of 50−100 nm was used with a dwell time of 30−60 ms to collect high‐resolution XRF maps. A photon‐counting Merlin detector (Quantum Detectors, UK) was also used in transmission configuration to collect X‐ray scattering for DPC images.

##### XAFS and Nano‐XANES Measurements

Reference materials were prepared as pellets and used for qualitative comparison and quantitative analysis of XAFS data (purified magnetosomes, Fe_3_O_4_, Fe_2_O_3_, 2‐line ferrihydrite, FeCl_2_, FeCl_3_). Enough iron oxide material was combined and mixed with cellulose (using pestle and mortar) to achieve one X‐ray absorption length. XAFS measurements of references were collected in transmission mode at the I‐14 nanoprobe using a photodiode detector. Nano‐XANES mapping was performed by collecting a series of XRF maps across the Fe K‐edge. To correct for drift during the measurement and to align the maps afterward, the Ti Kα XRF signal was used as a tracking and postalignment feature. Presented XAFS spectra from nano‐XANES mapping were smoothed using three‐point averaging (Figure 2 presents raw data and smoothed data for comparison).

##### TEM Analysis

TEM images were collected using a Tecnai G2 BioTWIN (FEI Company) electron microscope equipped with a charged‐coupled device (CCD) camera (Megaview III, Olympus Soft imaging Solutions GmbH) using an accelerating voltage of 100 kV.

##### Data and Image Analysis

ImageJ (Fiji version) was used to analyze TEM images and conduct particle size measurements. Athena program from the Demeter package was used to conduct XAFS data normalization, energy calibration, and LC fitting.^[^ ^37^ ^]^ A combinatorial approach was used for LC analysis by testing all combinations of reference materials (magnetite, hematite, goethite, ferrihydrite, ferric chloride, and ferrous chloride). The best fit was obtained by selecting the lowest number of references required to obtain a fit whose reduced‐χ^2^ value was not improved by 10% with an additional reference. When fitting cluster center spectra, the magnetite reference was omitted when the cluster was outside the magnetosome chain region. Dawn^[^ ^38^ ^]^ and Mantis^[^ ^39^ ^]^ software were used to interpret collected XRF maps and nano‐XANES dataset, with the latter used for principal components and cluster center analyses. Similarly, Python‐based scripts created by the I‐14 Nanoprobe group at Diamond Light Source were employed to perform normalization, alignment, and cluster analysis of nano‐XANES data. Silhouette scores/error maps were used to determine the minimum number of components required to describe the nano‐XANES dataset (limiting variance between cluster signals within its own group using the Cattell scree test, where the number of significant components was given by detecting an “elbow” feature in the eigenvalue plot).^[^ ^40^ ^]^

## Conflict of Interest

The authors declare no conflict of interest.

## Supporting information

Supplementary Material

## Data Availability

The data that support the findings of this study are available from the corresponding author upon reasonable request.
