# Supplementary material for: Synchrotron‐Based Nano‐X‐Ray Absorption Near‐Edge Structure Revealing Intracellular Heterogeneity of Iron Species in Magnetotactic Bacteria
Source: Small Sci. 2021 Dec 23;2(3):2100089. doi: 10.1002/smsc.202100089 (PMC11935880; doi:10.1002/smsc.202100089)
Supplement: Supplementary file 1 — Supplementary Material [file SMSC-2-2100089-s001.pdf]

Supporting Information

Synchrotron-based nano-XANES reveals intracellular heterogeneity of iron species in magnetotactic bacteria

Daniel M. Chevrier, Elisa Cerdá-Doñate, Yeseul Park, Fernando Cacho-Nerin, Miguel Gomez Gonzalez, René Uebe, Damien Faivre**

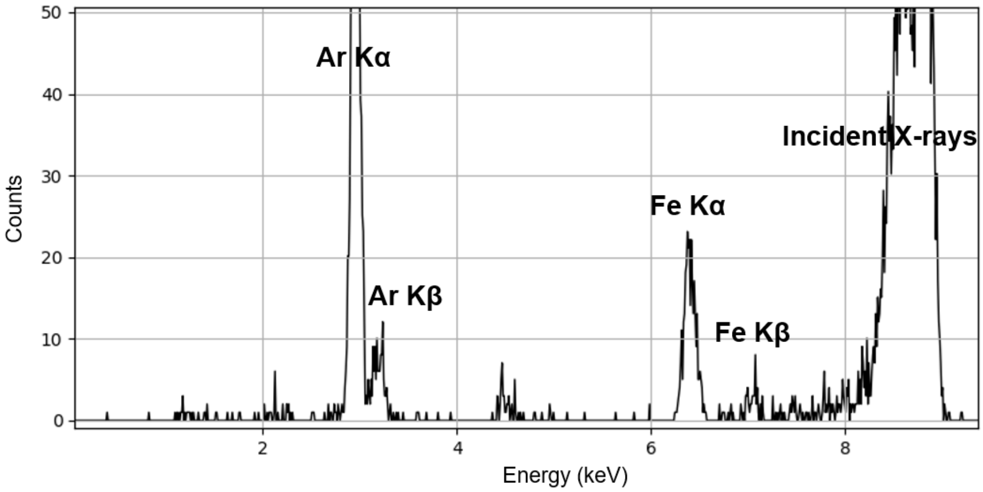

Figure S1. XRF spectrum from magnetosome chain region of MSR-1 *WT high iron* (Fig 1A).

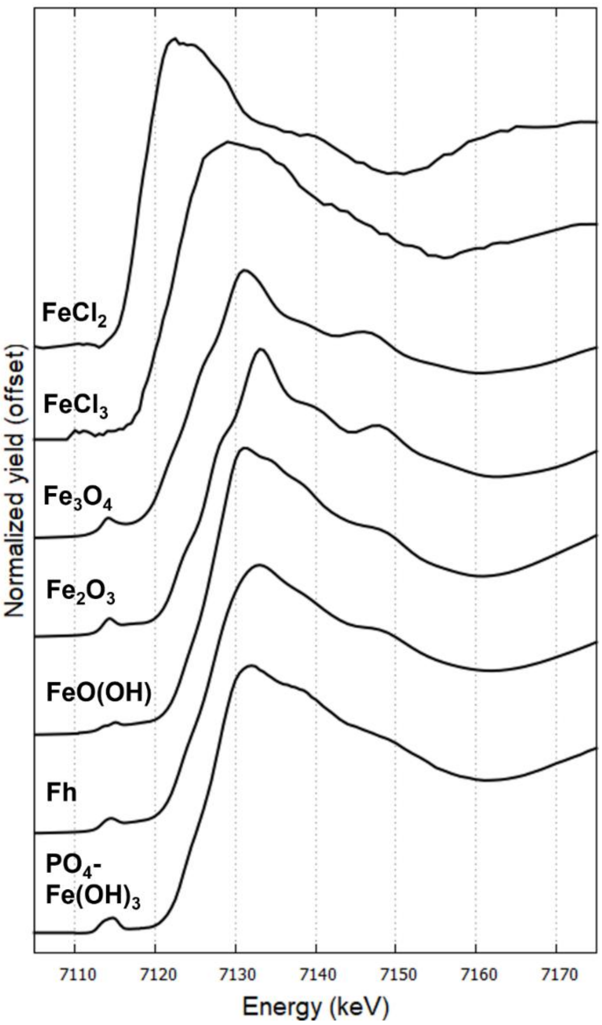

Figure S2. Fe K-edge XANES spectra of reference materials used in linear combination fitting (LCF). Fh – ferrihydrite, PO_4-$Fe(OH)_3$ – phosphate-rich ferric hydroxides.

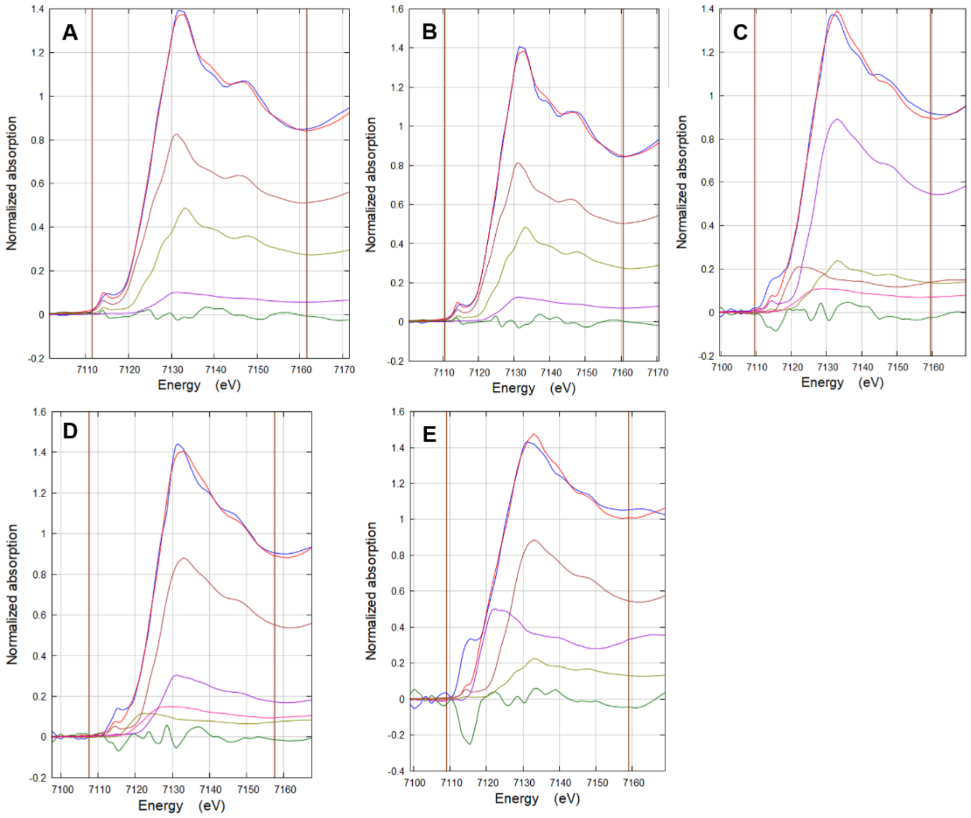

Figure S3. Linear combination fitting of *WT high iron* Fe K-edge XANES data for full cell region (A) and fitted cluster centers (cluster 1 (B), cluster 2 (C), cluster 3 (D) and cluster 4 (E)). Blue line – experimental data, red line – fit, green – residual from fit, vertical brown lines – fitting region.

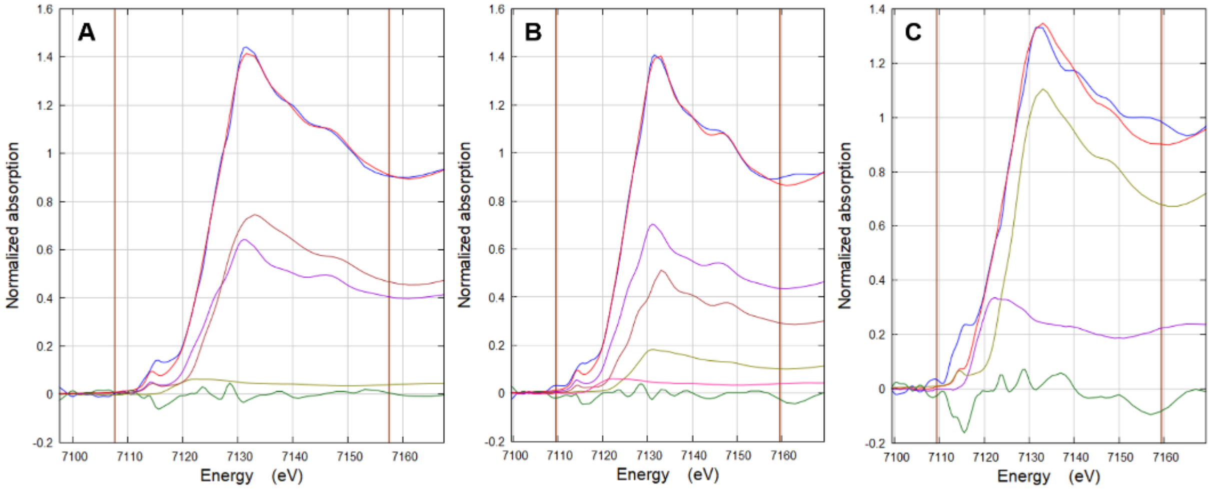

Figure S4. Linear combination fitting of *WT low iron* Fe K-edge XANES data for full cell region (A) and fitted cluster centers (cluster 1 (B) and cluster 2 (C)). Blue line – experimental data, red line – fit, green – residual from fit, vertical brown lines – fitting region.

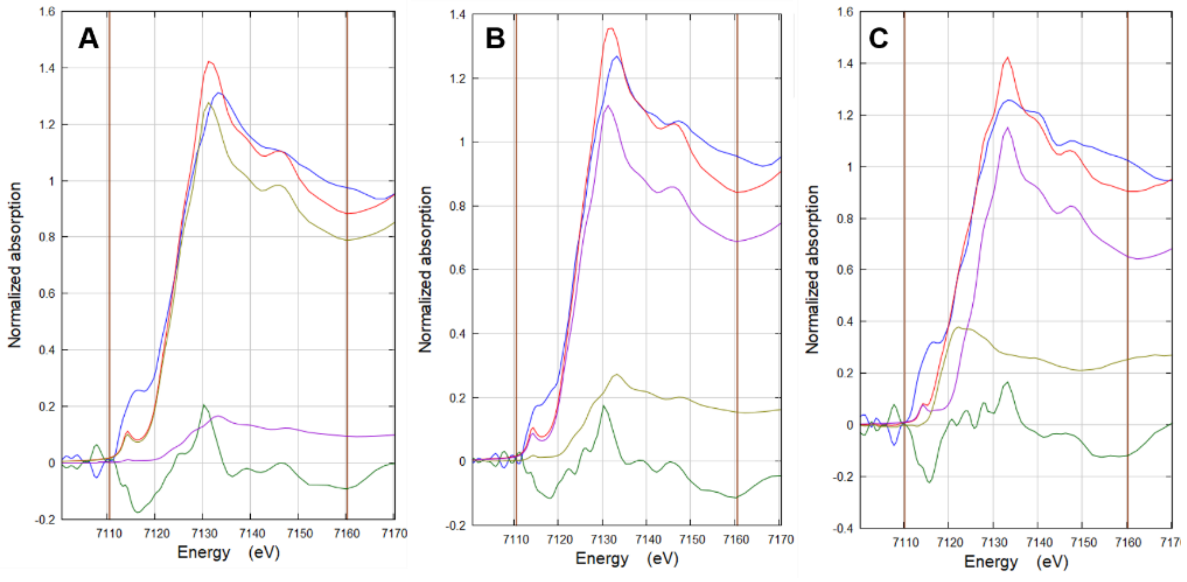

Figure S5. Linear combination fitting of *ΔmamB high iron 4 h* Fe K-edge XANES data for full cell region (A) and fitted cluster centers (cluster 1 (B) and cluster 2 (C)). Blue line – experimental data, red line – fit, green – residual from fit, vertical brown lines – fitting region.

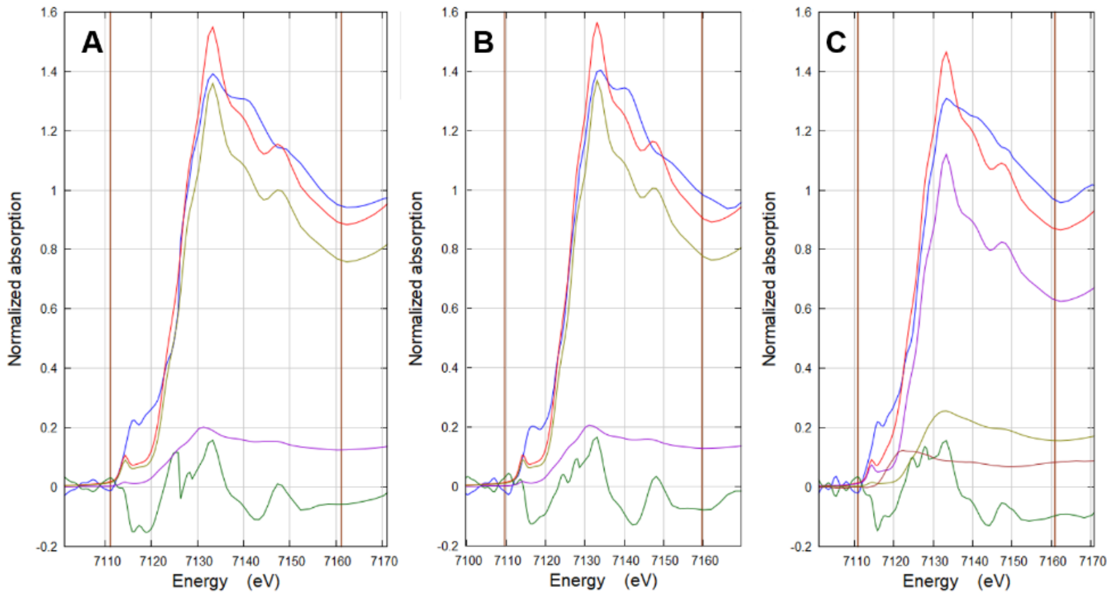

Figure S6. Linear combination fitting of *WT high iron 2 h* Fe K-edge XANES data for full cell region (A) and fitted cluster centers (cluster 1 (B) and cluster 2 (C)). Blue line – experimental data, red line – fit, green – residual from fit, vertical brown lines – fitting region.

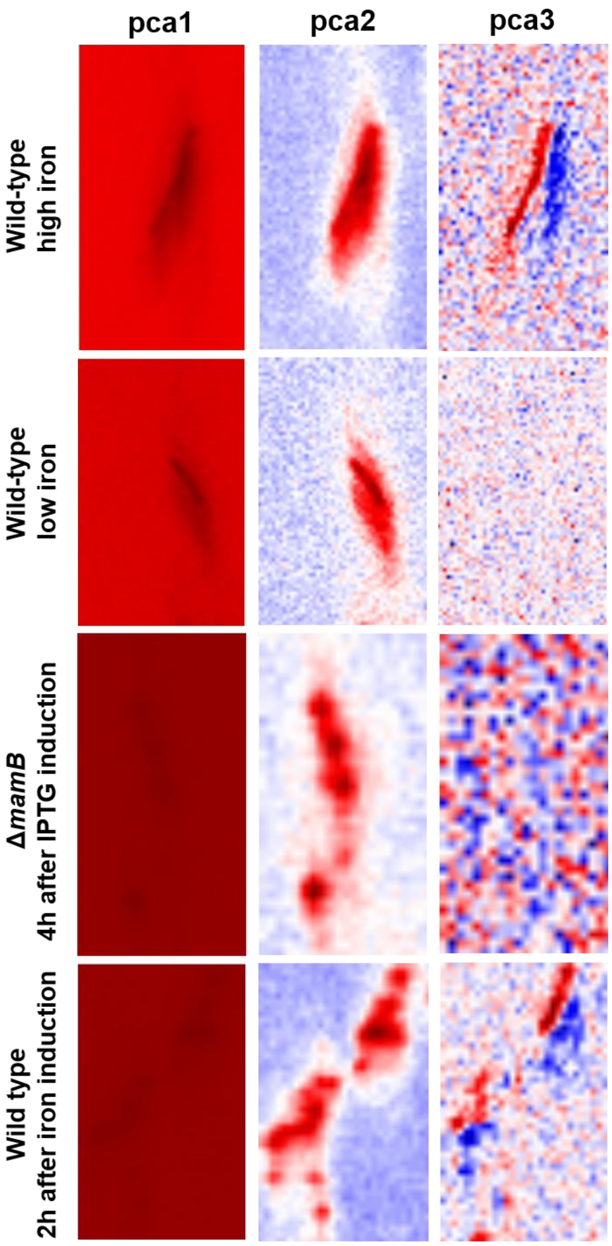

Figure S7. Principal components analysis maps or eigenimages for each sample.

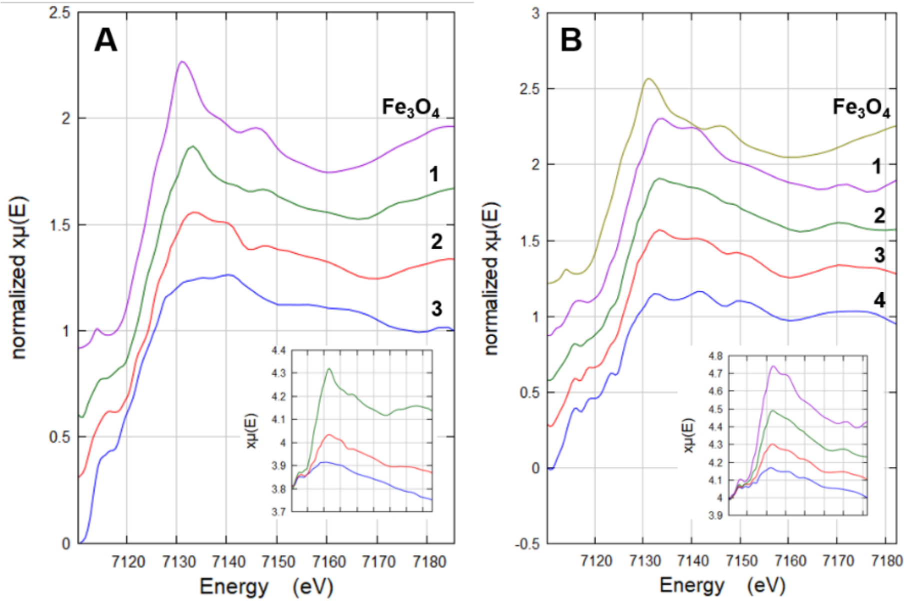

Figure S8. Fe K-edge XANES spectra for cluster centers with magnetite for (A) Δ*mamB* high iron 4 h and (B) WT high iron 2 h (inset with spectra retaining edge jump values)

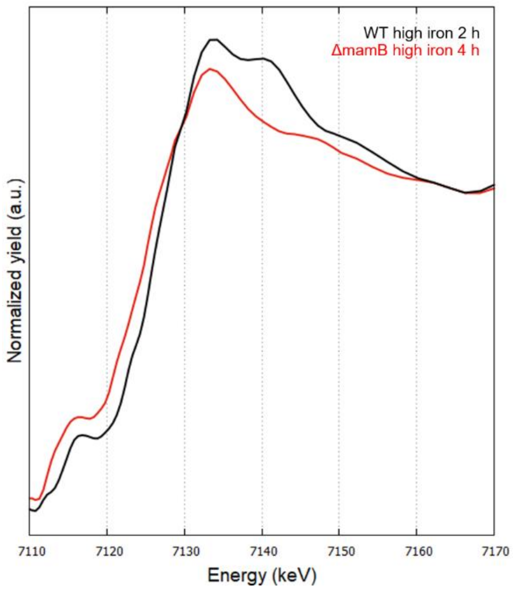

Figure S9. Fe K-edge XANES spectra of cluster 1 for *WT high iron 2 h* and *ΔmamB high iron 4 h.*

Table S1. List of samples investigated and respective growth conditions and magnetosome induction method.

Sample	50 µM Fe(III)-citrate added	Time (h) of extracted sample	Magnetosome formation induced
WT high iron	Yes	72	No
WT low iron	No	72	No
Δ*mamB* high iron 4 h	Yes	4	Yes (IPTG induction)
WT high iron 2 h	Yes	2	Yes (Iron induction)

Table S2. Linear combination fitting results of Fe K-edge XANES spectra from full cell and cluster centers. $\Delta E_{0\text{-}0mag} = E_{sample,0}\text{-}E_{magnetite,0}$ relative oxidation from magnetite reference.

Sample	Region	ΔE_0 (eV)	% Fe_3O_4	% Fe_2O_3	% Fh	% FeO(OH)	% $FeCl_3$	% $FeCl_2$	R-factor	red chi^2
**WT high iron**	**Full cell**	+1.0	60.0	33.2	0.0	6.8	0.0	0.0	0.00108	0.000233
	**1**	+1.1	58.7	32.8	0.0	8.5	0.0	0.0	0.00105	0.000240
	**2**	+0.2	0.0	16	64	0.0	7.0	13.0	0.00415	0.000921
	**3**	+0.1	0.0	0.0	62.8	20.2	9.7	7.3	0.00306	0.000836
	**4**	-	0.0	13.7	57.4	0.0	0.0	28.9	0.03234	0.007291
**WT low iron**	**Full cell**	+0.4	44.5	0.0	51.7	0.0	0.0	3.9	0.00173	0.000466
	**1**	+0.2	50.0	34.0	0.0	12.1	0.0	3.8	0.00155	0.000371
	**2**	+0.1	0.0	0.0	78.8	0.0	0.0	21.2	0.01819	0.003584
	**3**	-	-	-	-	-	-	-	-	-
**WT high iron 2 h**	**Full cell**	+1.6	13.8	86.2	0.0	0.0	0.0	0.0	0.03242	0.007783
	**1**	+1.8	13.9	86.1	0.0	0.0	0.0	0.0	0.02276	0.005989
	**2**	+1.4	0.0	74.1	18.1	0.0	0.0	7.8	0.03829	0.008258
	**3**	-	-	-	-	-	-	-	-	-
	**4**	-	-	-	-	-	-	-	-	-
**ΔmamB high iron 4 h**	**Full cell**	+1.0	89.2	10.8	0.0	0.0	0.0	0.0	0.04344	0.007915
	**1**	+1.0	81.4	18.6	0.0	0.0	0.0	0.0	0.02479	0.004577
	**2**	+0.7	0.0	76.2	0.0	0.0	0.0	23.8	0.05091	0.008981
	**3**	-	-	-	-	-	-	-	-	-
